# The predictive value of interleukin-2 receptor and prognostic nutritional index in patients with diffuse large B-cell lymphoma

**DOI:** 10.3389/fmed.2026.1737807

**Published:** 2026-01-27

**Authors:** Fengyang Xie, Yulan Li, Xinyu Zhu, Jingjing Zhu, Xiaoxia Ma, Dimei Yan, Aibin Liang, Bing Xiu

**Affiliations:** 1 Department of General Practice, Shanghai Jing’an District Caojiadu Community Health Service Center, Shanghai, China; 2 Department of Medical Imaging, Tongji Hospital, School of Medicine, Tongji University, Shanghai, China; 3 Department of Hematology, Tongji Hospital, School of Medicine, Tongji University, Shanghai, China; 4 Department of General Practice, Shanghai Baoshan Distric Dachang Community Health Service Center, Shanghai, China; 5 Department of General Practice, Tongji Hospital, School of Medicine, Tongji University, Shanghai, China; 6 Department of Hematology, Tongren Hospital, School of Medicine, Shanghai Jiao Tong University, Shanghai, China

**Keywords:** diffuse large B-cell lymphoma, IL-2R, immune status, prognosis, prognostic nutritional index

## Abstract

**Background:**

Diffuse large B-cell lymphoma (DLBCL) is the most common aggressive non-Hodgkin lymphoma (NHL), accounting for 30–40% of NHL cases. It exhibits high heterogeneity in gene expression and genetics, leading to significant variability in clinical treatment outcomes. Currently, various methods are available for predicting the prognosis of DLBCL patients, including the classic International Prognostic Index (IPI), as well as gene sequencing and circulating tumor DNA (ctDNA). However, some of these prognostic stratification methods are invasive and costly, limiting their widespread application. Therefore, there is an urgent need to identify potential prognostic indicators for lymphoma that can be widely used in the prognostic assessment of DLBCL patients, thereby further improving the stratification of DLBCL prognosis.

**Objective:**

This study aims to determine the prognostic value of serum interleukin-2 receptor (IL-2R) and prognostic nutritional index (PNI) in patients diagnosed with diffuse large B-cell lymphoma (DLBCL), as well as their applicability across different DLBCL subtypes.

**Methods:**

A retrospective analysis was conducted on 171 newly diagnosed DLBCL patients who received standard chemotherapy at Tongji Hospital in Shanghai from March 2013 to March 2024. Among them, 136 patients completed serum IL-2R testing. Spearman’s correlation analysis was used to describe the associations between different categorical indicators. The optimal cutoff values were determined based on receiver operating characteristic (ROC) curves. Kaplan-Meier analysis and log-rank tests were employed to compare survival rates among different subgroups. Finally, univariate and multivariate Cox proportional hazards regression models were applied to identify factors influencing the prognosis of DLBCL patients.

**Results:**

The baseline levels of IL-2R were correlated with patient age, nutritional status, and inflammatory response. PNI was associated with tumor burden in patients. Among the 136 patients, the cutoff value for IL-2R was 1,202 U/mL, while the cutoff value for PNI in the 171 patients was 44.65. Patients with high IL-2R and low PNI shared common characteristics, including advanced age, higher Ann Arbor stage, more frequent B symptoms, higher IPI scores, a higher proportion of intermediate-to-high-risk patients, poorer performance status, and shorter overall survival (OS) and progression-free survival (PFS). Multivariate analysis indicated that IL-2R > 1,202 U/mL and PNI ≤ 44.65 were independent risk factors for poor PFS and OS in newly diagnosed DLBCL patients.

## 1 Introduction

Diffuse large B-cell lymphoma (DLBCL) is a clinically and genetically heterogeneous disease originating from lymph nodes and other lymphoid tissues (1). Currently, DLBCL is considered a curable disease, with an average cure rate of approximately 70% (2). However, 30–40% of patients still experience treatment failure or relapse (3). Due to the heterogeneity of DLBCL, the application of various prognostic scoring systems in clinical practice has certain limitations. Some diagnostic methods, such as medical imaging, immunohistochemistry, flow cytometry, gene sequencing, and circulating tumor DNA (ctDNA), are partially invasive and impose a significant financial burden on patients. Therefore, it is necessary to explore clinically accessible indicators that can reflect disease progression and predict prognosis in DLBCL.

The expression of interleukin-2 receptor (IL-2R) is considered indicative of a persistent immune response in the body. Due to its relatively long half-life in the blood, IL-2R is easily detectable and is often used to assess immune-related diseases (4). As a heterotrimeric complex composed of CD25, CD122, and CD132 subunits, its soluble form (sIL-2R) is shed by activated lymphocytes and directly reflects tumor burden and immune microenvironment status in lymphoid malignancies (5, 6). Its prognostic utility stems from regulating regulatory T cell differentiation and immunosuppressive signaling, which are key to DLBCL progression (7). These characteristics make IL-2R a clinically actionable biomarker for risk stratification and treatment response monitoring in lymphoma patients. Notably, Ennishi D. et al. retrospectively analyzed 228 diffuse large B-cell lymphoma (DLBCL) patients (141 receiving R-CHOP, 87 receiving CHOP) and confirmed that high serum sIL-2R levels were strongly associated with poorer outcomes: in the R-CHOP group, 2-year event-free survival (EFS) was 66% vs. 92% and overall survival (OS) was 82% vs. 95% in high vs. low sIL-2R subgroups (both *P* < 0.01) (8). This prognostic value was further validated by Goto N et al. in 80 DLBCL patients treated with R-CHOP: elevated sIL-2R levels correlated with inferior OS (*P* = 0.015) and were closely linked to higher IPI scores and non-germinal center B-cell-like (non-GCB) subtypestwo well-established poor prognostic factors in DLBCL. Notably, sIL-2R also showed potential as a surrogate for IPI, offering a convenient, blood-based alternative for risk stratification in clinical practice (8, 9). Collectively, these studies confirm that sIL-2R retains robust prognostic value in the rituximab era, making it a clinically actionable biomarker for DLBCL patients undergoing standard R-CHOP therapy.

Prognostic nutritional index (PNI), another commonly used index calculated from lymphocytes and albumin levels, reflects both immune function and nutritional status. It has been widely applied in surgical risk assessment and prognosis evaluation of solid tumors (10, 11). In recent years, PNI has also demonstrated research value in hematology. A study of 435 cancer patients treated with immune checkpoint inhibitors (ICI) found that patients with higher PNI values had better prognoses (12).

Although IL-2R and PNI are widely used in clinical practice, their individual and combined roles in prognostic assessment for DLBCL remain unclear. As evidenced by the research background, IL-2R and PNI hold significant potential in hematological research and are easily accessible in clinical settings. Existing evidence primarily focuses on the independent predictive value of individual markers, and it remains unclear whether integrating IL-2R and PNI can further optimize the accuracy of risk stratification, or if it exerts consistent predictive efficacy across different subtypes (e.g., GCB/non-GCB) or clinical characteristics (e.g., elderly patients, advanced-stage disease). Therefore, this study aims to fill this gap by separately analyzing the independent prognostic significance of IL-2R and PNI, thereby laying a foundation for subsequent exploration of a more precise prognostic assessment model constructed by their combination, and ultimately providing clinical evidence f or individualized risk stratification and treatment decision-making in newly diagnosed DLBCL patients.

## 2 Materials and methods

### 2.1 Patients

A total of 245 newly diagnosed DLBCL patients who visited Tongji Hospital in Shanghai between March 2013 and March 2024 were retrospectively enrolled. Among them, 171 patients met the inclusion criteria. All patients were diagnosed according to the 2008 World Health Organization (WHO) classification of lymphoid neoplasms and its 2016 revision (13). Clinical staging was determined based on the extent of disease involvement, the presence or absence of B symptoms, and the Ann Arbor staging system. All patients before treatment were underwent routine examinations, including complete blood count, blood biochemistry, serum immunology, bone marrow morphology, lymph node ultrasound, chest and abdominal computed tomography (CT), and pathological immunohistochemistry. Some patients also underwent positron emission tomography-computed Tomography (PET-CT) and next-generation sequencing (NGS) for lymphoma gene mutations. We collected and recorded clinical information, survival time indicators, and treatment data during follow-up.

This study was approved by the hospital’s ethics review committee (Approval No. 2021-013-SK-XZ-210410). All procedures involving human participants were conducted in accordance with the Declaration of Helsinki (2013 revision). All participants were informed of the study’s purpose and content, and written informed consent was obtained from the participants or their authorized family members.

### 2.2 Inclusion criteria

(1)    Patients who were newly diagnosed and treated in the Hematology Department of our hospital from March 2013 to June 2023, met the classification and typing criteria for hematopoietic and lymphoid tissue tumors established by the World Health Organization (WHO), and were pathologically and immunohistochemically diagnosed as newly diagnosed DLBCL patients.
(2)    Patients who had completed at least 4 cycles of chemotherapy [rituximab, cyclophosphamide, doxorubicin, vincristine and prednisone (R-CHOP) or a regimen similar to R-CHOP, specifically including: R-EPOCH (rituximab, etoposide, prednisone, vincristine, cyclophosphamide, doxorubicin); R-CHOP-14 (R-CHOP administered every 14 days, a dose-dense regimen); DA-EPOCH-R (dose-adjusted EPOCH plus rituximab); R-CHOEP (R-CHOP plus etoposide, for high-risk DLBCL patients)].
(3)    Patients with complete clinical and laboratory data during the treatment and follow-up process.
(4)    Patients aged 16 years or older.

### 2.3 Exclusion criteria

(1)    Non-newly diagnosed DLBCL patients who had received chemotherapy, radiotherapy, or autologous stem cell transplantation before diagnosis.
(2)    Patients with other malignant tumor diseases.
(3)    Patients with severe comorbidities or organ dysfunction, specifically defined as: Cardiac insufficiency (New York Heart Association functional class III-IV); Liver function at Child-Pugh class C or severe coagulation disorders [International Normalized Ratio (INR) > 3.0]; Renal insufficiency [estimated glomerular filtration rate (eGFR) < 30 mL/min/1.73 m^2^]; Active severe infections (e.g., septicemia, uncontrolled pneumonia, etc.).
(4)    Patients lacking important initial diagnosis or follow-up data.

IL-2R: After admission, patients had their fasting venous blood drawn in the morning. After centrifugation to separate the serum, it was placed in the refrigerator for future use. The levels of IL-2R and others were measured by chemiluminescence (using the Siemens Immulete 1,000 analyzer, and the detection kit was purchased from Siemens).

PNI: PNI = Serum albumin (g/L) + 5 × Blood lymphocyte count (× 10^9^/L) (14).

### 2.4 Clinical assessment and follow-up

Follow-up of all patients was completed through methods such as case review, outpatient follow-up, inpatient re-examination, and telephone follow-up. The time points of disease progression, recurrence, or death of patients were recorded. During chemotherapy, follow-up was conducted every 3 weeks, and after discharge, it was carried out every 3 months. The criteria for efficacy evaluation refer to the modified Lugano Classification for Lymphoma (2014 version) (15), with specific definitions as follows:

(1)    Complete Remission (CR): All clinical and radiological lesions disappear, lymph nodes and masses shrink to normal size, or the sum of the products of the greatest diameters (SPD) decreases by > 75%, and tumor markers and biochemical indicators are within the normal range and remain stable for ≥ 4 weeks.
(2)    Partial Remission (PR): SPD decreases by > 50%, with no new lesions appearing and maintaining for ≥ 4 weeks.
(3)    Stable Disease (SD), neither PR nor progressive disease (PD).
(4)    PD: The size of any abnormal lymph nodes determined before treatment increases by > 50% compared to the previous minimum SPD value, or new lesions appear during or after treatment.

Survival data of patients were collected to calculate Overall Survival (OS) and Progression-Free Survival (PFS). OS is defined as the interval from admission to the trial to death from all causes, and PFS is defined as the interval from admission to the trial to disease progression or death. Both OS and PFS are measured in months. The follow-up time of all patients was not < 6 months, and the cut-off follow-up time was March 2024.

### 2.5 Statistical methods

Data analysis was performed using IBM SPSS statistical software (Version 23.0, IBM Inc., NY, United States) and GraphPad Prism (Version 8.0, GraphPad Software Inc., CA, United States). The Shapiro–Wilk test was used to examine whether continuous variables conformed to a normal distribution. For continuous variables with a normal distribution, the mean ± standard deviation was used for representation, while for those with a skewed distribution, the median was used. Categorical data were presented as frequencies or percentages. The Spearman correlation coefficient was used to represent the correlation between two variables. The receiver operating characteristic curve (ROC curve) was applied to determine the optimal cut-off values for IL-2R and PNI, respectively. When comparing baseline data between two groups, the *t*-test, Wilcoxon test, or Fisher’s test was employed. Survival analysis was carried out using the Cox proportional hazards model, with the hazard ratio as the evaluation index. The Kaplan–Meier method was used to plot survival curves. COX regression analysis was adopted to assess the relationship between various factors and prognosis. A *P-*value < 0.05 was considered statistically significant.

## 3 Results

### 3.1 Patient characteristics

**FIGURE 1:**
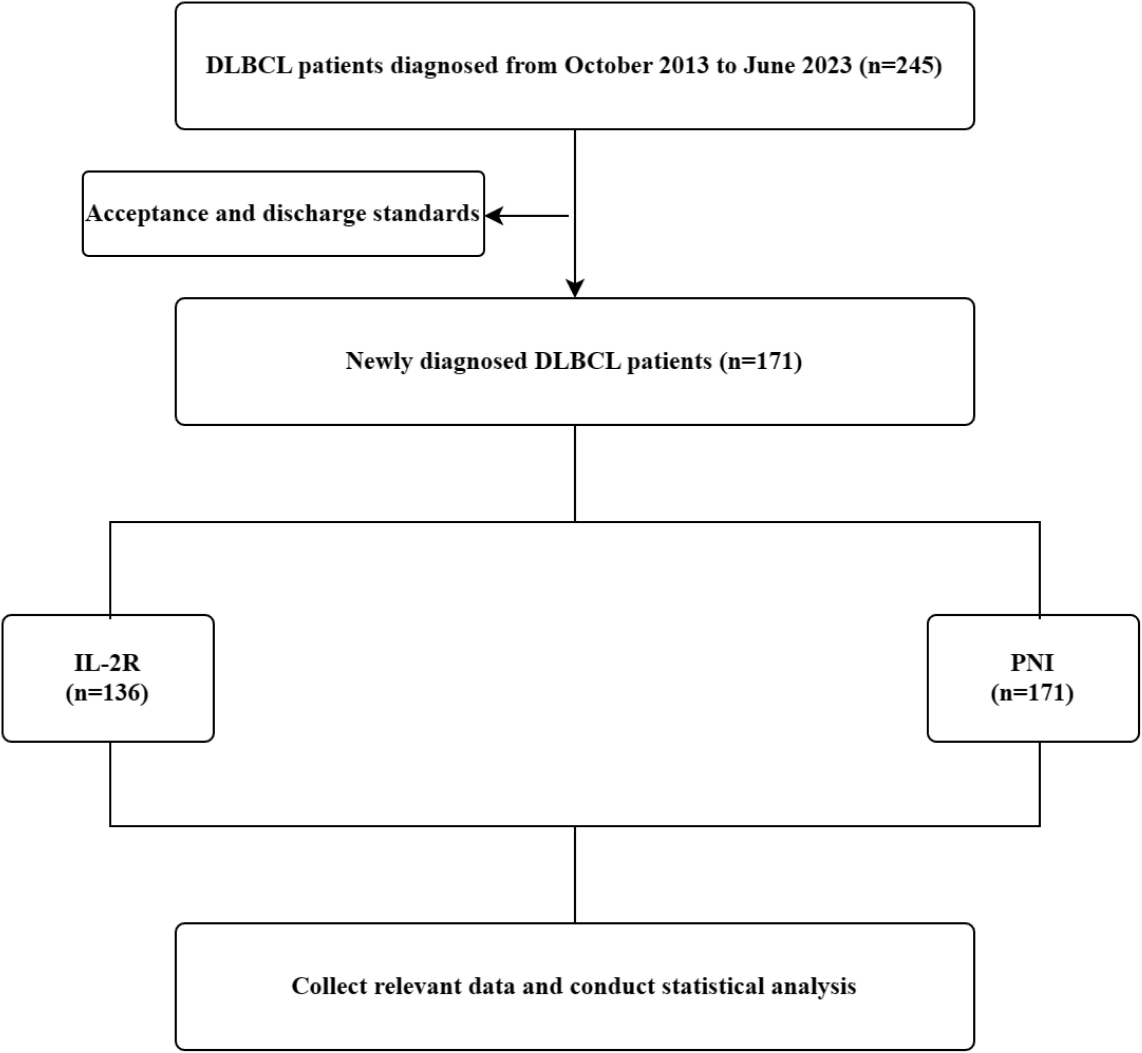
The screening process for IL-2R and PNI research. IL-2R, interleukin-2 receptor; PNI, prognostic nutritional index.

From Oct 2013 to Jun 2023, 245 DLBCL patients were diagnosed. After screening by specific criteria, 171 newly-diagnosed patients were enrolled and divided into IL-2R (*n* = 136) and PNI (*n* = 171) groups. Relevant data was collected for statistical analysis to deepen DLBCL research (see Figure 1). Up to the final follow-up time of March 2024 for the observation, the median follow-up time was 54.33 months. Among the patients, 108 (63.2%) achieved complete remission (CR) after receiving at least 4 courses of treatment, while 63 (36.8%) did not achieve CR. The overall survival rate was 69%. Detailed information on patient characteristics is provided in Table 1.

**TABLE 1:** Characteristics of 171 patients with DLBCL.

Characteristics		n(%)
Gender	Male	101(59.1)
	Female	70(40.9)
Age (years)	≤ 60	73(42.7)
	>60	98(57.3)
B symptoms	Absence	88(51.5)
	Presence	83(48.5)
ECOG score	<2	105(61.4)
	≥ 2	66(38.6)
Ann Arbor stage	I–II	74(43.3)
	III–IV	97(56.7)
IPI score	≤ 2	90(52.6)
	>2	81(47.4)
Risk stratification	Low risk	38(22.2)
	Low intermediate risk	38(22.2)
	High intermediate risk	48(28.1)
	High risk	47(27.5)
Bone marrow invasion	Yes	21(12.3)
	No	150(87.7)
Cell origin	GCB	62(36.3)
	non-GCB	109(63.7)
BMI	Low weight	13(7.6)
	Normal weight	120(70.2)
	Overweight and obesity	38(22.2)
Ki-67(%)	<80	71(41.5)
	≥ 80	100(58.5)
LDH(U/L)	<250	90(52.6)
	≥ 250 U/L	81(47.4)
ß2-MG(mg/L)	>2.8	69(40.4)
	≤ 2.8 mg/L	102(59.6)
Ferritin(ng/mL)	>400	77(45)
	≤ 400 ng/mL	94(55)
Hb(g/L)	≤ 115	74(43.3)
	>115 g/L	97(56.7)
ALB(g/L)	<40	125(73.1)
	≥ 40 g/L	46(26.9)
Follow-up after treatment	CR	108(63.2)
	PR	4(2.3)
	SD	4(2.3)
	PD	2(1.2)
	DIE	43(25.1)
	Loss to follow-up	10(5.8)

ECOG PS score, Eastern Cooperative Oncology Group performance status score. IPI, International Prognostic Index; GCB, generation center B-cell; non-GCB, non-germinal center B-cell; BMI, body mass index; LDH, lactic dehydrogenase; ß2-MG, ß2-microglobulin; Hb, hemoglobin; ALB, albumin; CR, complete response; PR, partial response; SD, stable disease; PD, progression disease.

### 3.2 Significant associations of IL-2R and PNI with key clinical characteristics in DLBCL patients

**FIGURE 2:**
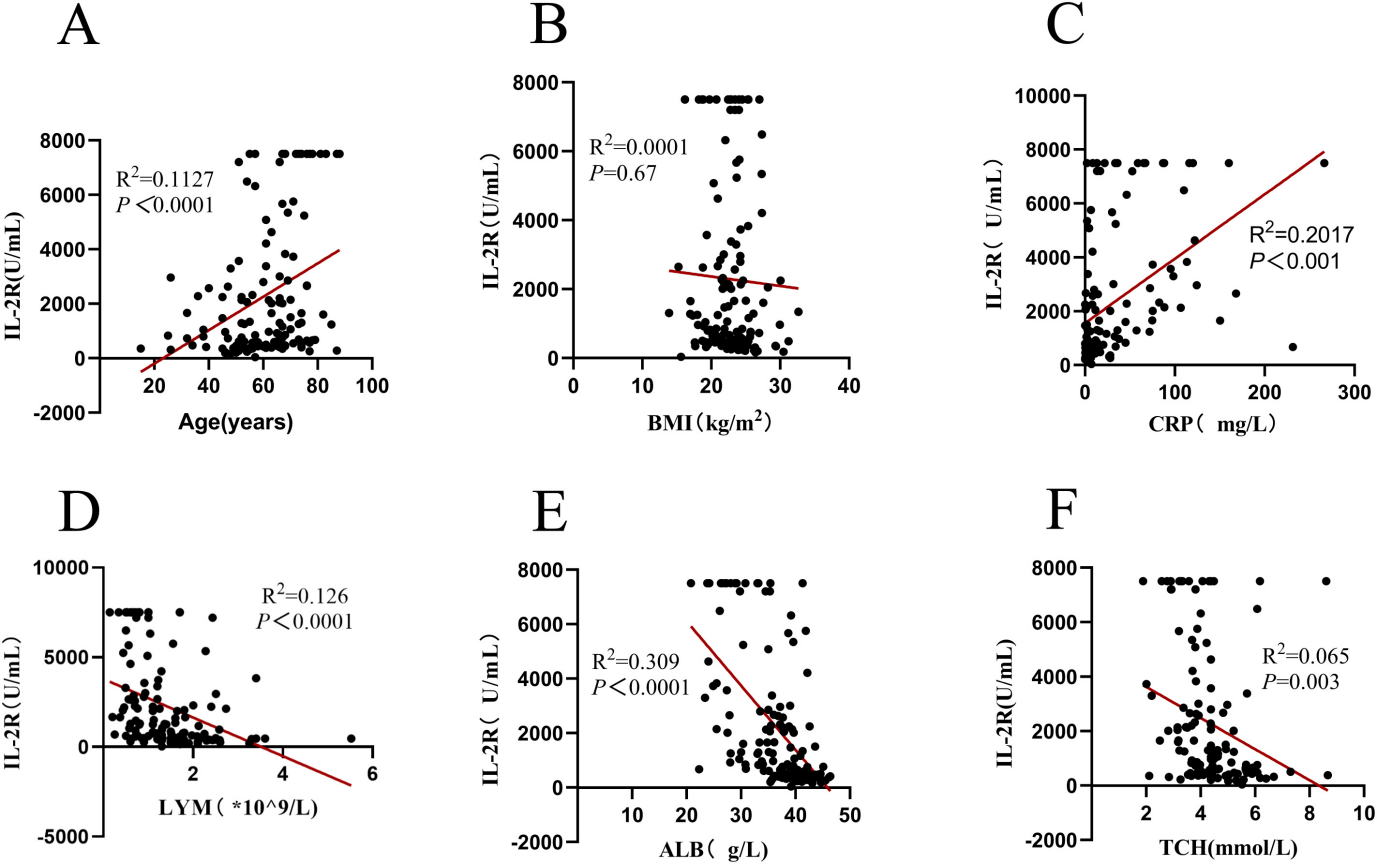
Correlation analysis of IL-2R in 136 DLBCL patients. **(A)** Correlation between IL-2R and age (*R*^2^ = 0.1127, *P* < 0.0001); **(B)** correlation between IL-2R and BMI (*R*^2^ = 0.0001, *P* = 0.67); **(C)** correlation between IL-2R and CRP (*R*^2^ = 0.2017, *P* < 0.001); **(D)** correlation between IL-2R and LYM (*R*^2^ = 0.126, *P* < 0.0001); **(E)** Correlation between IL-2R and ALB (*R*^2^ = 0.309, *P* < 0.0001); **(F)** correlation between IL-2R and TCH (*R*^2^ = 0.065, *P* = 0.003). IL-2R, interleukin-2 receptor; BMI, body mass index; CRP, C-reactive protein; LYM, lymphocyte; ALB, albumin; TCH, total cholesterol.

To explore the relationships between IL-2R, PNI and different types of clinical indicators, Spearman’s correlation coefficient analysis was performed. IL-2R was strongly correlated with age, C-Reactive Protein (CRP), lymphocyte count, albumin level, and total cholesterol (TCH) (all *P* < 0.05), but had no correlation with body mass index (BMI) (see Figure 2).

**FIGURE 3:**
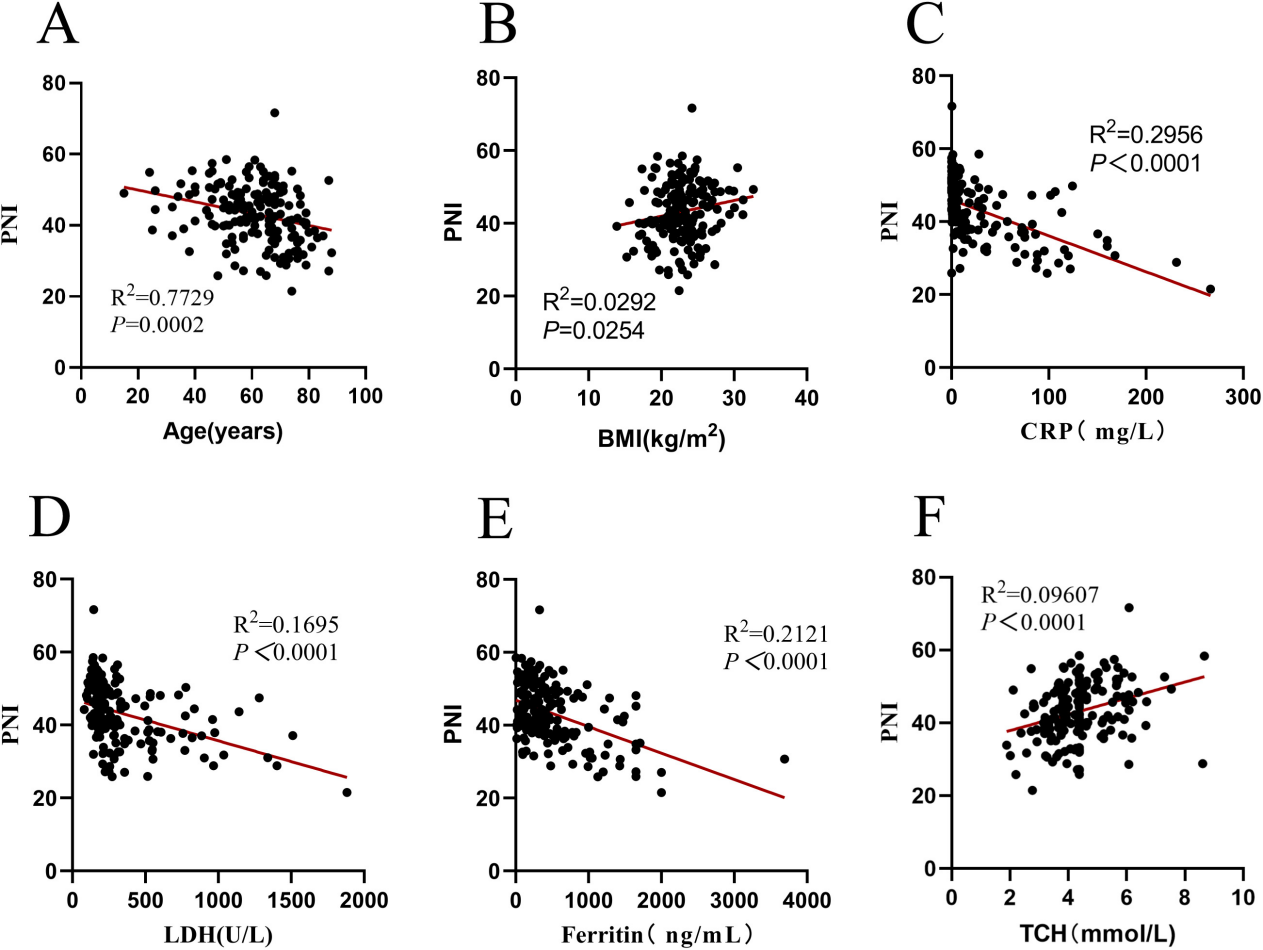
Correlation analysis of PNI in 171 DLBCL patients. **(A)** Correlation between PNI and age (*R*^2^ = 0.7729, *P* = 0.0002); **(B)** correlation between PNI and BMI (*R*^2^ = 0.0292, *P* = 0.0254); **(C)** correlation between PNI and CRP (*R*^2^ = 0.2956, *P* < 0.0001); **(D)** correlation between PNI and LDH (*R*^2^ = 0.1695, *P* < 0.0001); **(E)** Correlation between PNI and ferritin (*R*^2^ = 0.2121, *P* < 0.0001); **(F)** correlation between PNI and TCH (*R*^2^ = 0.09607, *P* < 0.0001). PNI, prognostic nutritional index; BMI, body mass index; CRP, C-reactive protein; LDH, lactic dehydrogenase; TCH, total cholesterol.

Since PNI is composed of lymphocyte count and albumin. It is inevitable that they have a correlation, that’s the reason why this study did not explore the relationships between PNI, lymphocytes, and albumin again. In the research on PNI, PNI was correlated with the age, BMI, CRP, lactic dehydrogenase (LDH), ferritin, and TCH of DLBCL patients (all *P* < 0.05) (see Figure 3 for details).

In summary, IL-2R is closely linked to immune and nutritional markers (lymphocytes, albumin) and systemic inflammation (CRP), while PNI correlates with inflammatory indicators (CRP, LDH, ferritin) and age, reflecting their potential as integrated markers of disease-related status.

### 3.3 Optimal cut-off values of IL-2R and PNI for prognostic stratification in DLBCL patients

**FIGURE 4:**
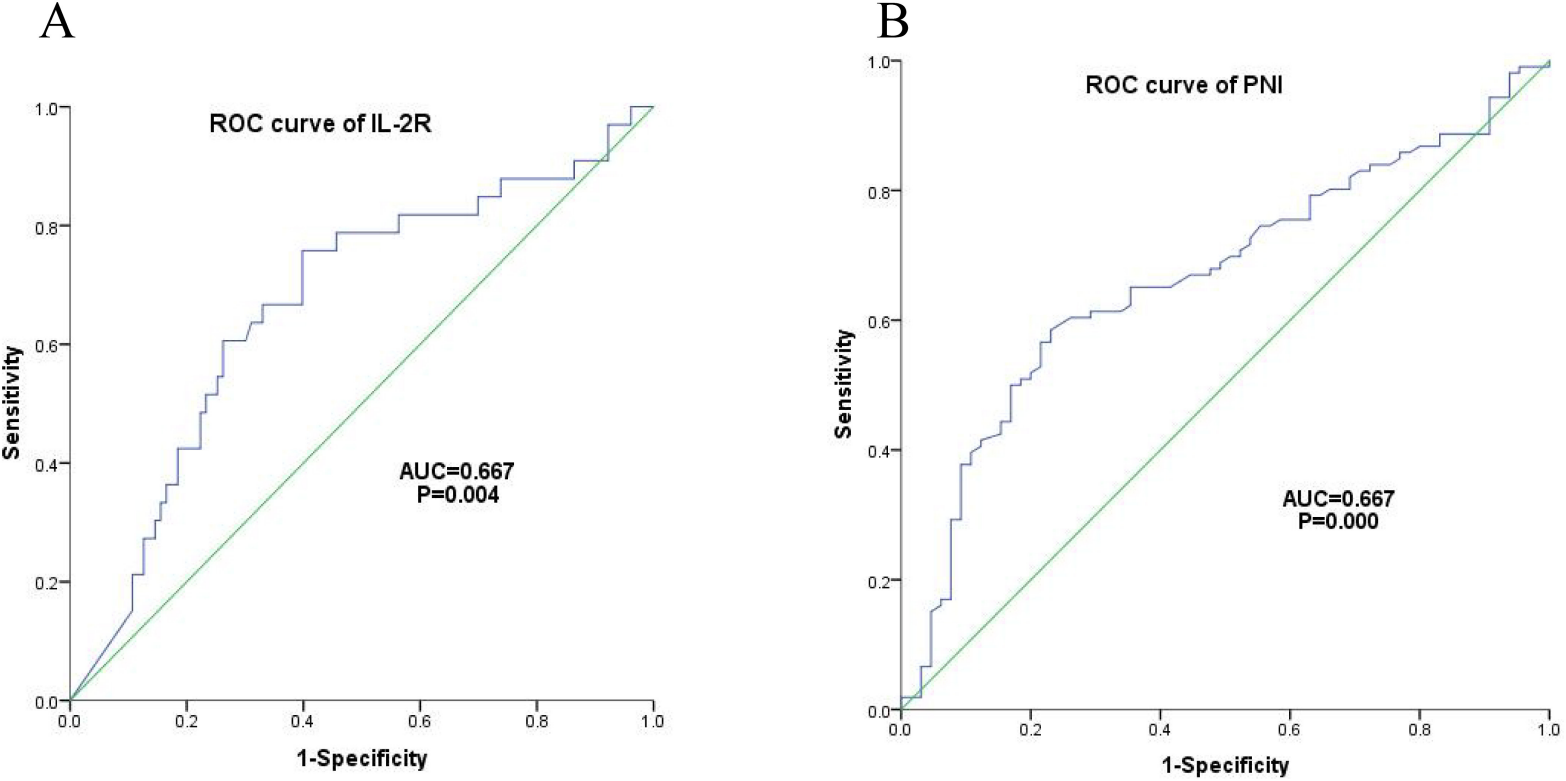
Optimal cut-off values of IL-2R. **(A)** ROC curve of IL-2R, cut-off value = 1,202 U/mL, sensitivity = 0.758, specificity = 0.602, AUC = 0.667 (95% CI: 0.559–0.776), *P* = 0.004; **(B)** ROC curve of PNI, cut-off value = 44.65, sensitivity = 0.585, specificity = 0.769, AUC = 0.667 (95% CI: 0.585–0.750), *P* < 0.001. IL-2R, interleukin-2 receptor; PNI, prognostic nutritional index; ROC, receiver operating characteristic; AUC, area under the curve.

There were 136 DLBCL patients in the IL-2R cohort. According to the ROC curve, the optimal cut-off value of IL-2R was calculated to be 1,202 U/mL, with a sensitivity of 0.758, a specificity of 0.602, an area under the curve (AUC) of 0.667 (95% CI: 0.559–0.776, *P* = 0.004). Among the 171 patients who were fully included, the optimal cut-off value of PNI calculated from the ROC curve was 44.65, with a sensitivity of 0.585, a specificity of 0.769, an area under the curve of 0.667 (95% CI: 0.585–0.750, *P* < 0.001) (see Figure 4 for details).

### 3.4 Distinct clinical characteristics between high/low IL-2R and PNI subgroups in DLBCL patients

Among 136 patients stratified by IL-2R cutoff value (1,202 U/mL), significant differences were observed in key clinical characteristics between the low and high IL-2R groups (all *P* < 0.05). The high IL-2R group had a higher proportion of patients aged > 60 years (68.2% vs. 47.1%), Ann Arbor stage III-IV (81.8% vs. 40.0%), presence of B symptoms (71.2% vs. 27.1%), IPI score 3–5 (69.7% vs. 28.6%), high-risk stratification (42.4% vs. 12.9%), ECOG score ≥ 2 (50.0% vs. 30.0%), and bone marrow invasion (22.7% vs. 4.3%). No significant differences were found in gender, cell origin, Ki-67 expression, or BMI between the two groups (all *P* > 0.05). The specific research results are shown in Table 2.

**TABLE 2:** Clinical characteristics of IL-2R subgroup DLBCL patients.

Characteristics	Total (*n* = 136)	IL-2R ≤ 1,202 U/mL (*n* = 70)	IL-2R >1,202 U/mL (*n* = 66)	*P*
Gender				0.362
Male	77(56.6%)	37(52.9%)	40(60.6%)	
Female	59(43.4%)	33(41.7%)	26(39.4%)	
Age(years)				0.013*
≤ 60	58(42.6%)	37(52.9%)	33(47.1%)	
>60	78(57.4%)	33(47.1%)	45(68.2%)	
Ann Arbor stage				< 0.001*
I–II	54(39.7%)	42(60.0%)	12(18.2%)	
III–IV	82(60.3%)	28(40.0%)	54(81.8%)	
B symptoms				< 0.001*
Absence	70(51.5%)	51(72.9%)	19(28.8%)	
Presence	66(48.5%)	19(27.1%)	47(71.2%)	
IPI score				< 0.001*
1–2	70(51.5%)	50(71.4%)	20(30.3%)	
3–5	66(48.5%)	20(28.6%)	46(69.7%)	
Risk stratification				< 0.001*
Low risk	26(19.1%)	24(34.3%)	2(3.0%)	
Low intermediate risk	32(23.5%)	18(25.7%)	14(21.2%)	
High intermediate risk	41(30.1%)	19(27.1%)	22(33.3%)	
High risk	37(27.2%)	9(12.9%)	28(42.4%)	
ECOG score				0.017*
<2	82(60.3%)	49(70.0%)	33(50.0%)	
≥ 2	54(39.7%)	21(30.0%)	33(50.0%)	
Cell origin				0.770
Non-GCB	89(65.4%)	45(64.3%)	44(66.7%)	
GCB	47(34.6%)	25(35.7%)	22(33.3%)	
Ki-67				0.063
<80%	59(43.4%)	25(35.7%)	34(51.5%)	
≥ 80%	77(56.6%)	45(64.3%)	32(48.5%)	
Bone marrow invasion				0.002*
No	118(86.8%)	67(95.7%)	51(77.3%)	
Yes	18(13.2%)	3(4.3%)	15(22.7%)	
Extranodal invasion				0.965
No	27(19.9%)	14(20.0%)	13(19.7%)	
Yes	109(80.1%)	56(80.0%)	53(80.3%)	
BMI				0.819
Normal weight	70(51.5%)	49(70.0%)	45(68.2%)	
Abnormal weight	66(48.5%)	21(30.0%)	21(31.8%)	

*Representative *P* < 0.05.

The clinical characteristics of DLBCL patients in the PNI subgroup were analyzed in a larger sample cohort of 171 people. In this cohort patients divided by PNI cutoff value (44.65), the low PNI group showed distinct clinical features compared with the high PNI group (all *P* < 0.05). Patients with PNI ≤ 44.65 were more likely to be aged > 60 years (64.9% vs. 47.3%), have Ann Arbor stage III-IV (70.1% vs. 29.9%), present with B symptoms (59.8% vs. 33.8%), IPI score 3–5 (58.8% vs. 32.4%), high-risk stratification (37.1% vs. 14.9%), ECOG score ≥ 2 (47.4% vs. 27.0%), and Ki-67 ≥ 80% (51.5% vs. 67.6%). Gender, cell origin, bone marrow invasion, and BMI did not differ significantly between the two groups (all *P* > 0.05). The detailed statistical results are shown in Table 3.

**TABLE 3:** Clinical characteristics of PNI subgroup DLBCL patients.

Characteristics	Total (*n* = 171)	PNI ≤ 44.65 (*n* = 94)	PNI>44.65 (*n* = 77)	*P*
Gender				0.685
Male	101(59.1%)	56(57.7%)	45(60.8%)	
Female	70(40.9%)	41(42.3%)	29(39.2%)	
Age(years)				0.021*
≤ 60	73(42.7%)	34(35.1%)	39(52.7%)	
>60	98(57.3%)	63(64.9%)	35(47.3%)	
Ann Arbor stage				< 0.001*
I–II	74(43.3%)	29(29.9%)	45(60.8%)	
III–IV	97(56.7%)	68(70.1%)	29(29.9%)	
B symptoms				0.001*
Absence	88(51.5%)	39(40.2%)	49(66.2%)	
Presence	83(48.5%)	58(59.8%)	25(33.8%)	
IPI score				0.001*
1–2	90(52.6%)	40(41.2%)	50(67.6%)	
3–5	81(47.4%)	57(58.8%)	24(32.4%)	
Risk stratification				0.001*
Low risk	38(22.2%)	12(12.4%)	26(35.1%)	
Low intermediate risk	22(22.2%)	22(22.7%)	16(21.6%)	
High intermediate risk	48(28.1%)	27(27.8%)	21(28.4%)	
High risk	47(27.5%)	36(37.1%)	11(14.9%)	
ECOG score				0.007*
<2	105(61.4%)	51(52.6%)	54(73.0%)	
≥ 2	66(38.6%)	46(47.4%)	20(27.0%)	
Cell origin				0.486
Non-GCB	109(63.7%)	64(66.0%)	45(60.8%)	
GCB	62(36.3%)	33(34.0%)	29(39.2%)	
Ki-67				0.035*
<80%	71(41.5%)	47(48.5%)	24(32.4%)	
≥ 80%	100(58.5%)	50(51.5%)	50(67.6%)	
Bone marrow invasion				0.967
No	150(87.7%)	85(87.6%)	65(87.8%)	
Yes	21(12.3%)	12.4(12%)	9(12.2%)	
Extranodal invasion				0.146
No	33(19.3%)	15(15.5%)	18(24.3%)	
Yes	138(80.7%)	82(84.5%)	56(75.7%)	
BMI				0.718
Normal weight	120(70.2%)	67(69.1%)	53(71.6%)	
Abnormal weight	51(29.8%)	30(30.9%)	21(28.4%)	

*Representative *P* < 0.05.

From this, we can conclude that High IL-2R and low PNI are consistently associated with adverse clinical features (advanced stage, high-risk IPI, B symptoms) in DLBCL, indicating high levels of IL-2R and low PNI are associated with disease progression in DLBCL patients.

### 3.5 Prognostic value of IL-2R and PNI for overall and progression-free survival in DLBCL patients

**FIGURE 5:**
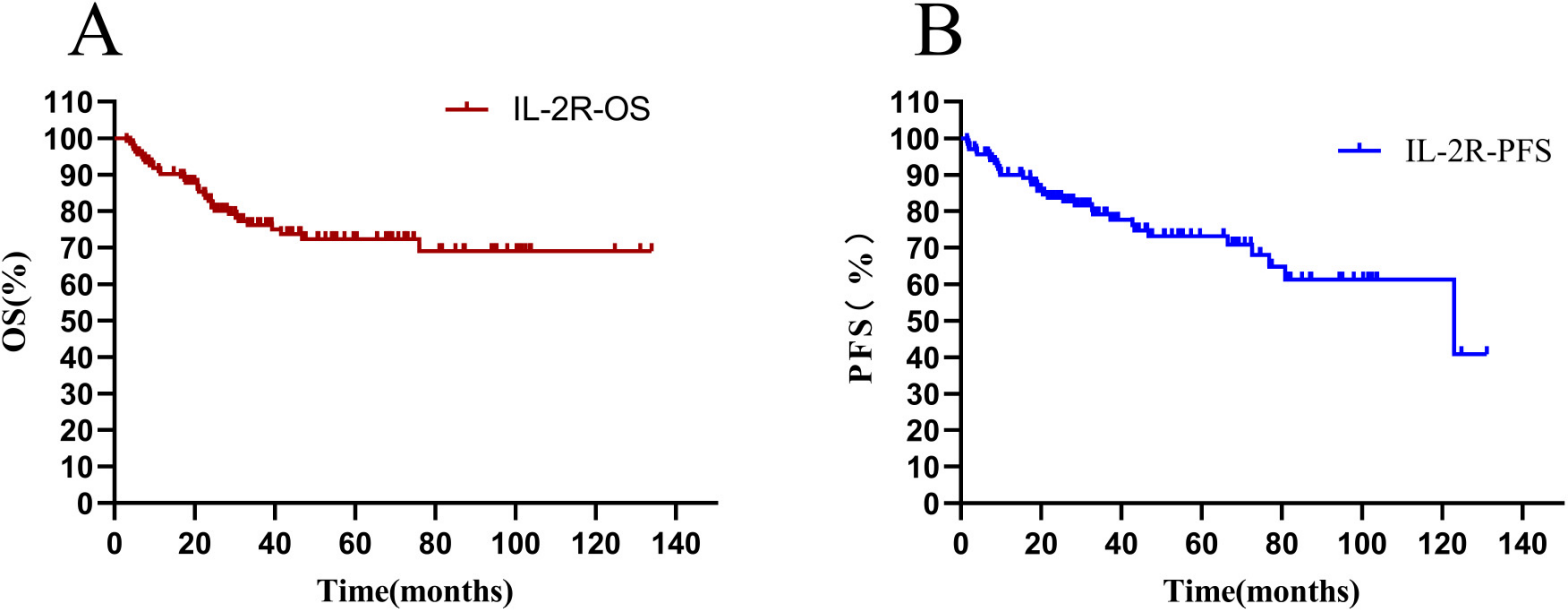
Total survival analysis of patients with IL-2R. **(A)** Overall survival curve of patients in the IL-2R group; **(B)** progression-free survival curve of patients in the IL-2R group. OS, overall survival; PFS, progression-free survival; IL-2R, interleukin-2 receptor.

The overall survival rate of 136 newly diagnosed DLBCL patients was 65.44%, and the median OS time was 36.10 (20.80–67.86) months (see Figure 5A). The progression—free survival rate was 72.06%, and the median PFS time was 31.29 (17.33–64.00) months (see Figure 5B).

**FIGURE 6:**
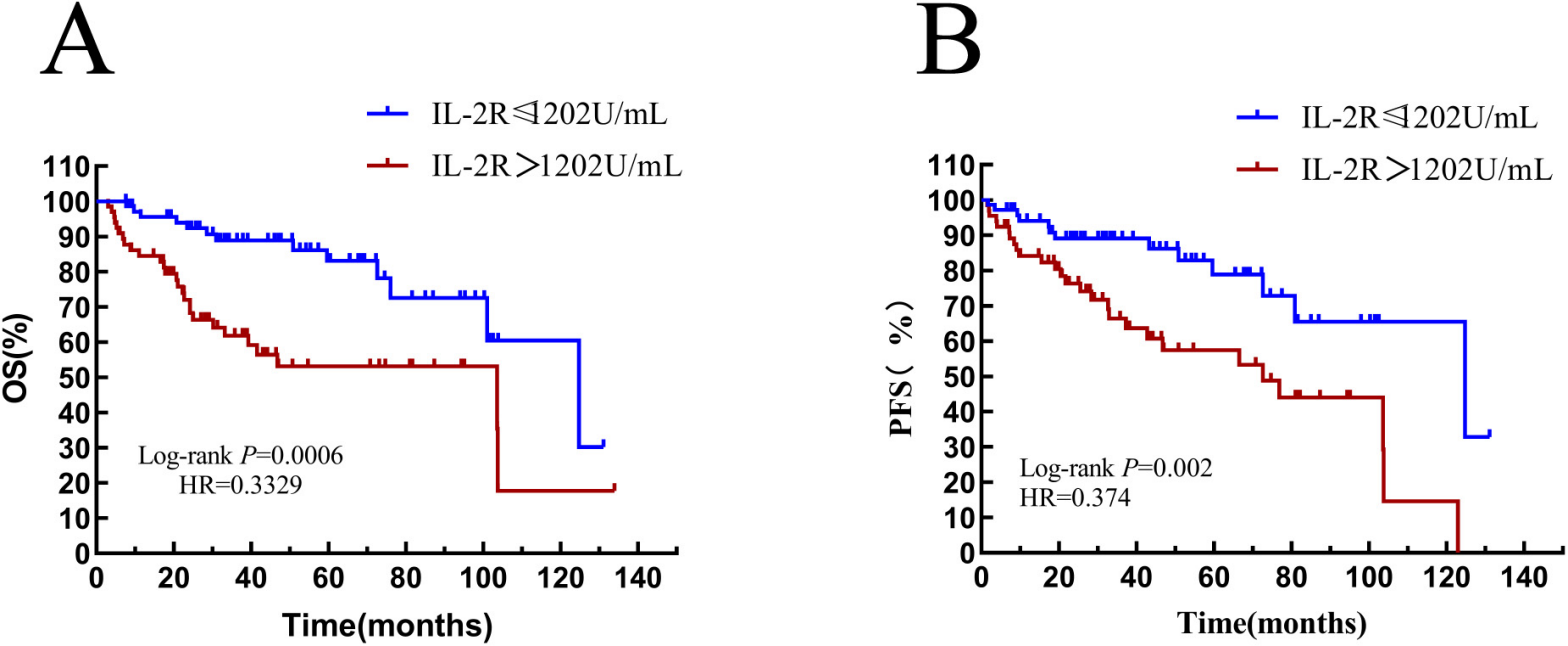
Overall survival and progression-free survival in patients stratified by IL-2R. **(A)** Overall survival curves of patients in IL-2R groups; **(B)** progression-free survival curves of patients in IL-2R groups. OS, overall survival; PFS, progression-free survival; IL-2R, interleukin-2 receptor.

The differences in survival rate and progression—free survival rate between the low-IL-2R and high-IL-2R groups were compared. The median OS time of patients with IL-2R ≤ 1,202 U/mL was 47.35 (HR = 0.33, 95% CI: 0.621–2.33) months, and that of patients with IL-2R > 1,202 U/mL was 27.53 (HR = 3.00, 95% CI: 1.559–5.752) months. There was a statistically significant difference between them (HR = 0.3329, *P* = 0.0006) (see Figure 6A). The median PFS time of patients with IL-2R ≤ 1,202 U/mL was 34.33 (HR = 0.37, 95% CI: 0.200–0.699) months, and that of patients with IL-2R > 1,202 U/mL was 27.54 (HR = 2.673, 95% CI: 1.431–4.993) months. There was a statistically significant difference between them (HR = 0.374, *P* = 0.002) (see Figure 6B).

**FIGURE 7:**
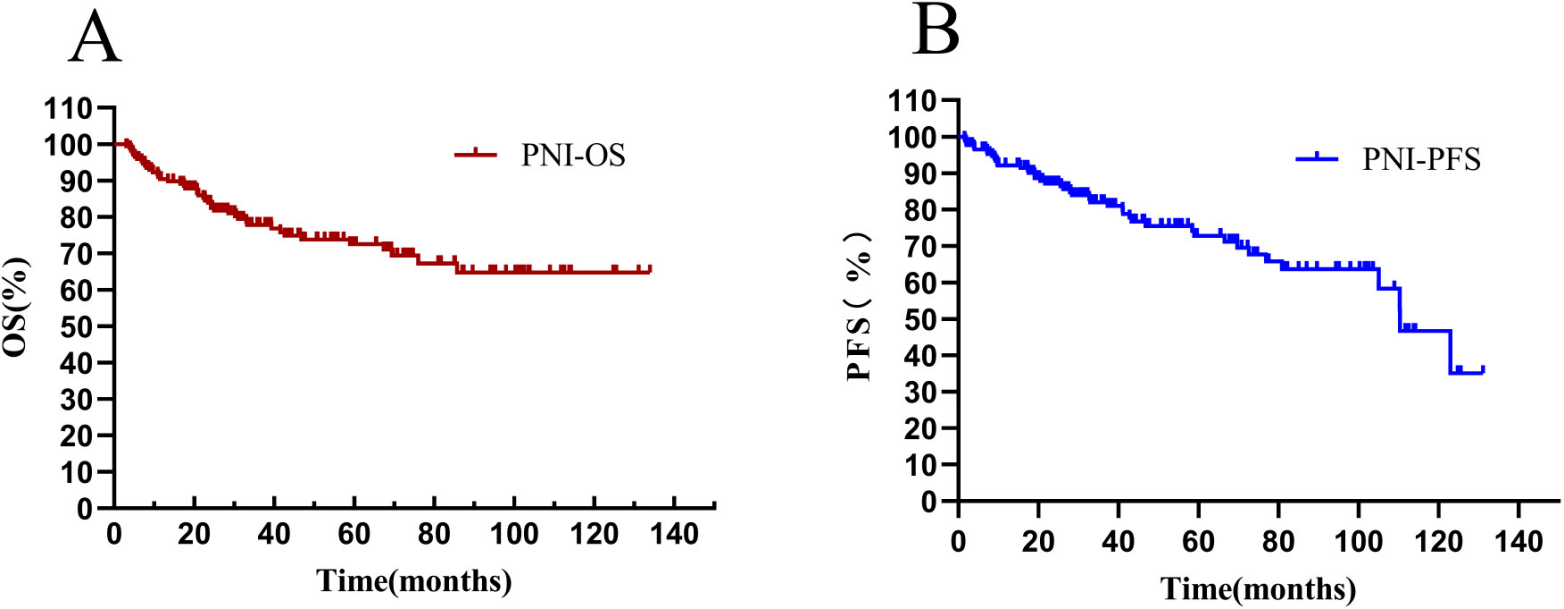
Total survival analysis of patients with PNI. **(A)** Overall survival curve of patients in the PNI group; **(B)** progression-free survival curve of patients in the PNI group. OS, overall survival. PFS, progression-free survival; PNI, prognostic nutritional index.

The overall survival rate of 171 newly-diagnosed DLBCL patients in the whole group was 69.01%, and the median OS time was 36.1 (20.80–67.87) months (see Figure 7A). The progression-free survival rate was 75.44%, and the median PFS time was 33.83 (18.80–69.67) months (see Figure 7B).

**FIGURE 8:**
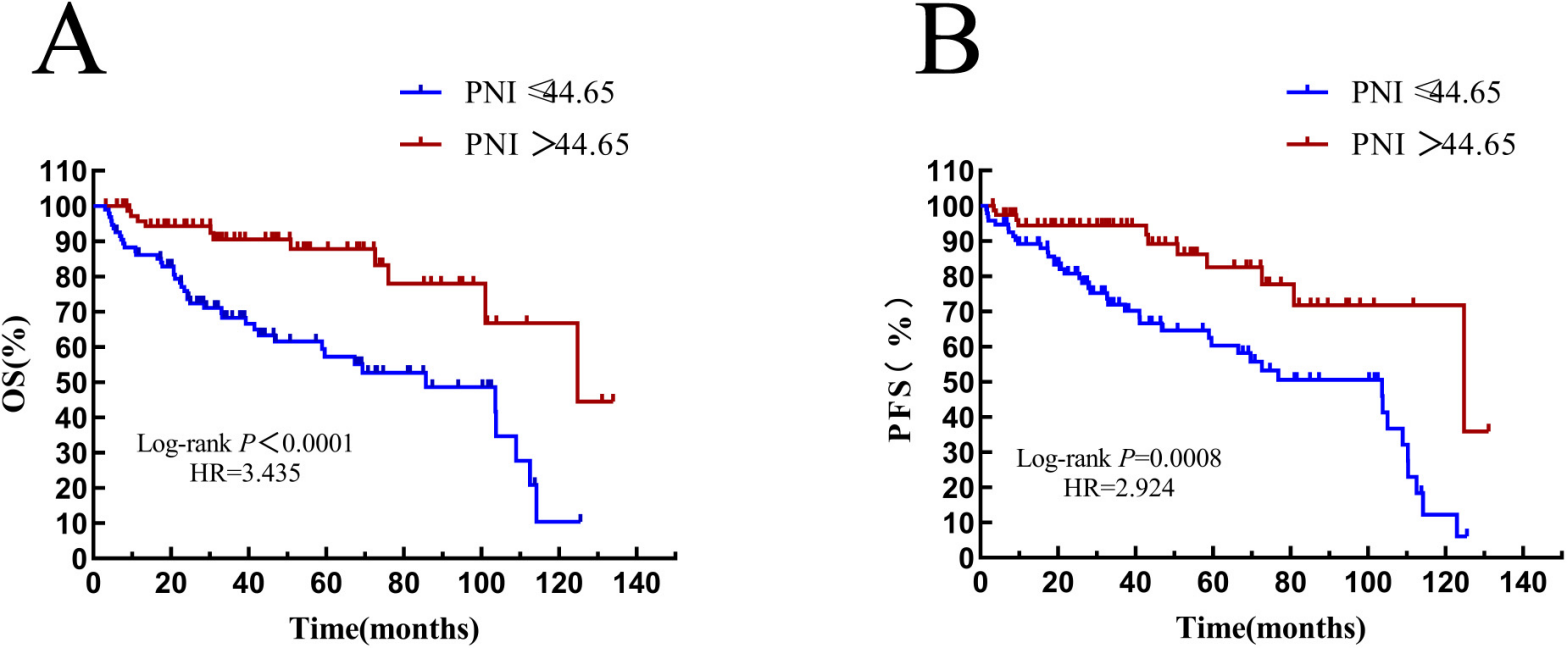
Overall survival and progression-free survival in patients stratified by PNI. **(A)** Overall survival curves of patients in PNI groups; **(B)** progression-free survival curves of patients in PNI groups. OS, overall survival; PFS, progression-free survival; IL-2R, interleukin-2 receptor.

The median OS time of patients in the PNI ≤ 44.65 group was 34.1 (HR = 0.6871, 95% CI: 0.3538–1.335) months, and that of patients with PNI > 44.65 was 39.2 (HR = 1.455, 95% CI: 0.7493–2.872) months. There was a statistically significant difference between them (HR = 3.435, *P* < 0.0001) (see Figure 8A). The median PFS time of patients with PNI ≤ 44.65 was 31.85 (HR = 0.8306, 95% CI: 0.4276–1.613) months, and that of patients with PNI > 44.65 was 37.83 (HR = 1.204, 95% CI: 0.6199–2.338) months. The result was statistically significant (HR = 2.924, *P* = 0.0008) (see Figure 8B).

**FIGURE 9:**
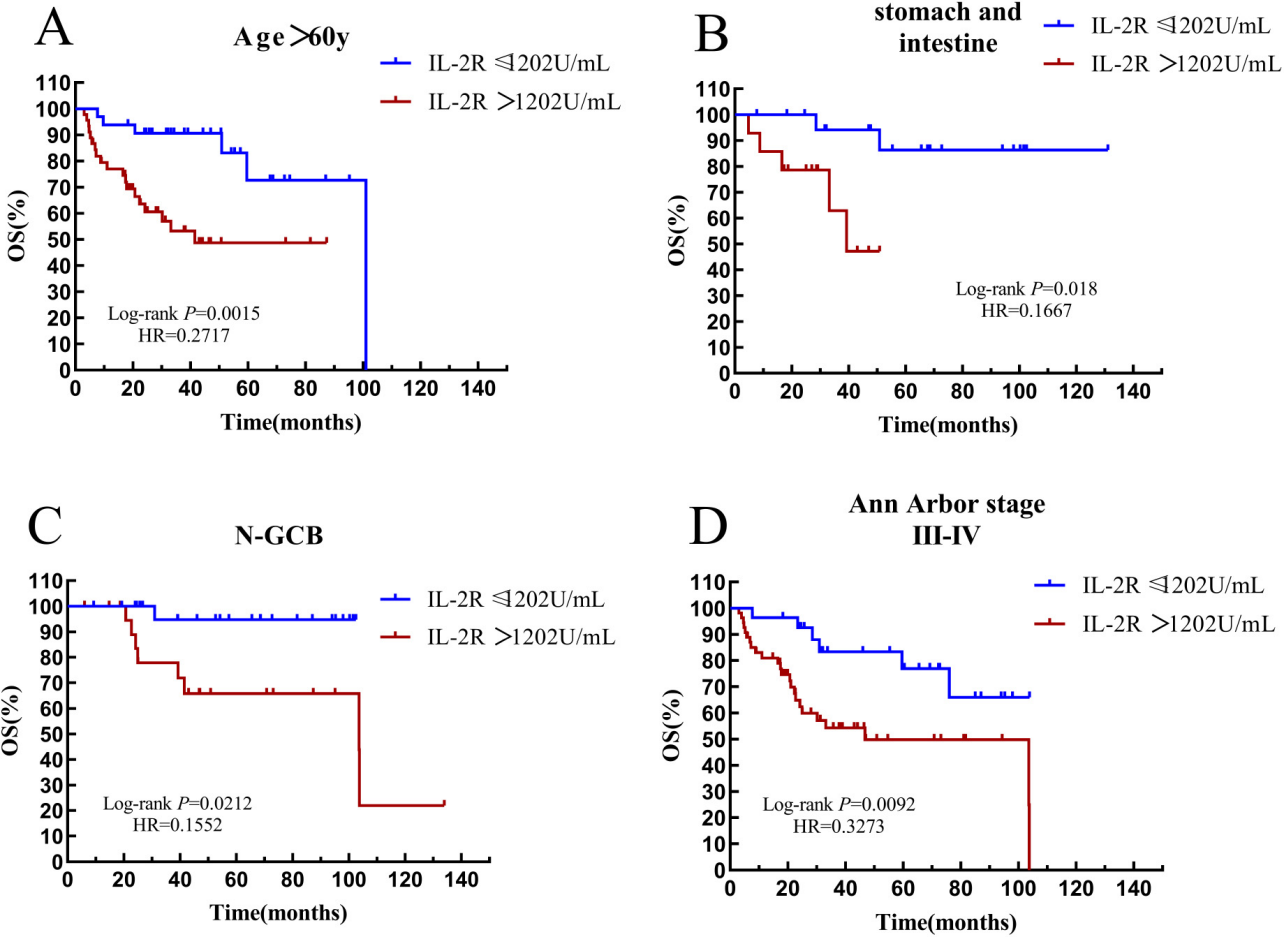
Survival analysis of the IL-2R subgroup. **(A)** The subgroup with age > 60 years old. **(B)** The subgroup with gastrointestinal tract. **(C)** The subgroup with non-GCB. **(D)** The subgroup with Ann Arbor stage III-IV. OS, overall survival; PFS, progression-free survival; IL-2R, interleukin-2 receptor; N-GCB, non-germinal center B-cell.

Patients with an age > 60 years old were defined as the elderly subgroup. In the IL-2R cohort, there were 78 patients in the elderly group. Among them, 33 patients had IL-2R ≤ 1,202 U/mL, with a median OS time of 39.1 (HR = 2.434, 95% CI: 0.9720–6.094) months, and 45 patients had IL-2R > 1,202 U/mL, with a median OS time of 22.33 (HR = 0.4109, 95% CI: 0.1641–1.029) months. The overall survival prognosis of the low—IL-2R group was better than that of the high-IL-2R group, and the difference was statistically significant (*P* = 0.0015) (see Figure 9A).

According to the site of onset, patients with lesions in the stomach and intestine were defined as the gastrointestinal subgroup. In the IL-2R cohort, there were 34 patients in the gastrointestinal group. Among them, 20 patients had IL-2R ≤ 1,202 U/mL, and the median OS time was not reached. 14 patients had IL-2R > 1,202 U/mL, with a median OS time of 27.9 months. The overall survival of the low-IL-2R group was better than that of the high-IL-2R group (*P* = 0.018) (see Figure 9B).

In DLBCL, the non-germinal center B-cell-like (non-GCB) subtype indicates a poor prognosis. In the IL-2R cohort, there were 47 patients in the non-GCB subgroup. Among them, 25 patients had IL-2R ≤ 1,202 U/mL, and the median OS time was not reached. 22 patients had IL-2R > 1,202 U/mL, with a median OS time of 42.28 months. In the non-GCB subgroup, the overall survival prognosis of the low-IL-2R group was better than that of the high-IL-2R group, and the difference was statistically significant (*P* = 0.0212) (see Figure 9C).

Patients with stage III–IV were listed as a subgroup. In the IL-2R cohort, there were 82 patients in the III–IV stage subgroup. Among them, 28 patients had IL-2R ≤ 1,202 U/mL, and the median OS time was not reached. 54 patients had IL-2R > 1,202 U/mL, with a median OS time of 22.68 months. In the III–IV stage subgroup, the overall survival prognosis of the low-IL-2R group was better than that of the high-IL-2R group, and the difference was statistically significant (*P* = 0.0062) (see Figure 9D).

**FIGURE 10:**
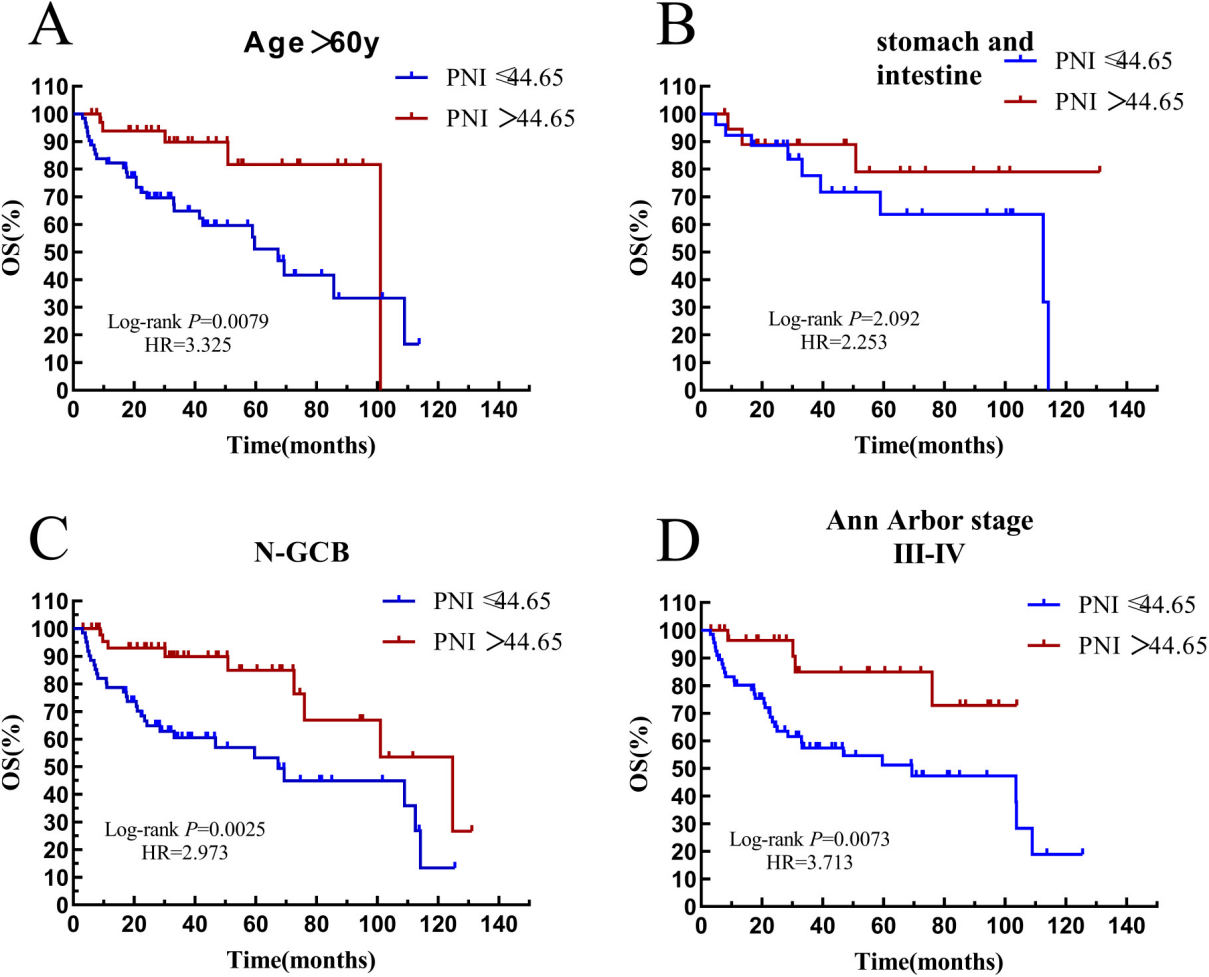
Survival analysis of the PNI subgroup. **(A)** The subgroup with age > 60 years old. **(B)** The subgroup with gastrointestinal tract. **(C)** The subgroup with non-GCB. **(D)** The subgroup with Ann Arbor stage III-IV. OS, overall survival; PFS, progression-free survival; PNI, prognostic nutritional index; N-GCB, non-germinal center B-cell.

Among the 171 patients, 98 patients were over 60 years old. In the elderly subgroup, 62 patients had a PNI ≤ 44.65, with a median survival time of 31.44 (HR = 0.6670, 95% CI: 0.2576–1.727) months, and 36 patients had a PNI > 44.65, with a median survival time of 36.07 (HR = 1.499, 95% CI: 0.5789–3.883) months. The result was statistically significant (*P* = 0.0079) (see Figure 10A).

There were 45 patients with the disease in the stomach and intestine tract in the whole group. In this subgroup, 26 patients had a PNI ≤ 44.65, with a median survival time of 36.24 months, and 19 patients had a PNI > 44.65, and the median survival time was not reached. There was no significant difference in the overall survival rate between the two groups (*P* = 0.2092) (see Figure 10B).

There were 109 patients with the non-GCB subtype in the whole group. In the non-GCB subgroup, 61 patients had a PNI ≤ 44.65, with a median survival time of 29.13 (HR = 0.5400, 95% CI: 0.2564–1.137) months, and 48 patients had a PNI > 44.65, with a median survival time of 35.35 (HR = 1.852, 95% CI: 0.8793–3.901). The prognostic difference between the two groups was statistically significant (*P* = 0.0007) (see Figure 10C).

There were 97 patients with stage III–IV in the whole group. In the III–IV stage subgroup, 66 patients had a PNI ≤ 44.65, with a median survival time of 29.92 months, and 31 patients had a PNI > 44.65, and the median survival time was not reached. The result was statistically significant (*P* = 0.0073) (see Figure 10D).

Through survival analysis, we confirmed that low IL-2R and PNI are associated with significantly improved OS and PFS in DLBCL, with consistent prognostic value across subgroups, with consistent prognostic value across key subgroups (elderly, non-GCB, advanced stage), affirming the consistency of their prognostic performance.

### 3.6 IL-2R and PNI are independent prognostic factors for overall and progression-free survival in newly diagnosed DLBCL patients

To further investigate whether IL-2R and PNI affect the prognostic survival of patients, univariate and multivariate analyses were conducted separately for the IL-2R subgroup and the PNI subgroup. The results are as follows.

In 136 patients with DLBCL, univariate analysis showed that Ann Arbor stage, B symptoms, ECOG, bone marrow involvement, LDH level, IL-2R level, and IPI were factors influencing the overall survival of DLBCL patients. Further multivariate analysis showed that gender, cell origin, IL-2R, and IPI (all *P* < 0.05) were independent prognostic factors for overall survival in DLBCL patients, as shown in Table 4.

**TABLE 4:** Univariate and multivariate analysis (OS) of prognostic factors in 136 patients with DLBCL.

Variables	Univariate(OS)		Multivariate(OS)	
	HR(95%CI)	*P*	HR(95%CI)	*P*
Gender	0.522(0.248–1.098)	0.087	0.366(0.162–0.830)	0.016*
Age	1.970(0.949–4.091)	0.096		
Ann Arbor stage	3.778(1.558–9.156)	0.001		
B symptoms	2.114(1.049–4.261)	0.036		
ECOG score	2.279(1.141–4.549)	0.019		
Cell origin	0.411(0.444–2.984)	0.771	0.308(0.114–0.834)	0.020*
Bone marrow invasion	1.152(0.178–1.949)	0.037		
Extranodal invasion	0.505(0.240–1.061)	0.071		
LDH	3.452(1.603–7.433)	0.001		
IL-2R	4.365(1.964–9.698)	< 0.001	3.288(1.281–8.440)	0.013*
IPI	3.939(1.842–8.507)	< 0.001	2.636(1.159–5.995)	0.021*

*Representative *P* < 0.05.

A study on progression-free survival in 136 patients with DLBCL showed that univariate analysis identified age, Ann Arbor stage, ECOG score, cell origin, extranodal involvement, LDH level, IL-2R level, and IPI as factors influencing PFS in DLBCL patients. Multivariate analysis indicated that gender, cell origin, IL-2R, and IPI were independent risk factors for disease progression (all *P* < 0.05), as shown in Table 5.

**TABLE 5:** Univariate and multivariate analysis (PFS) of prognostic factors in 136 patients with DLBCL.

Variables	Univariate(PFS)		Multivariate(PFS)	
	HR(95%CI)	*P*	HR(95%CI)	*P*
Gender	0.511(0.242–1.077)	0.067	0.336(0.144–0.783)	0.012*
Age	2.208(1.053–4.631)	0.036		
Ann Arbor stage	3.982(1.638–9.680)	0.002		
B symptoms	1.929(0.955–3.894)	0.067		
ECOG score	2.166(1.085–4.326)	0.027		
Cell origin	0.411(0.177–0.954)	0.039	0.277(0.095–0.803)	0.018*
Bone marrow invasion	1.073(0.412–2.797)	0.885		
Extranodal invasion	0.434(0.203–0.927)	0.031		
LDH	3.400(1.577–7.329)	0.001		
IL-2R	3.934(1.772–8.753)	0.001	3.044(1.151–8.050)	0.025*
IPI	4.102(1.899–8.858)	< 0.001	2.609(1.115–6.102)	0.027*

*Representative *P* < 0.05.

A study on the overall survival of 171 patients with DLBCL showed that univariate analysis indicated gender, age, Ann Arbor stage, ECOG, LDH level, PNI, and IPI were factors affecting the OS of 171 newly-diagnosed DLBCL patients. Multivariate analysis showed that gender, PNI, and IPI (all *P* < 0.05) were independent prognostic factors for the OS of DLBCL, as shown in Table 6.

**TABLE 6:** Univariate and multivariate analysis (OS) of prognostic factors in 171 patients with DLBCL.

Variables	Univariate(OS)		Multivariate(OS)	
	HR(95%CI)	*P*	HR(95%CI)	*P*
Gender	0.476(0.256–0.883)	0.019	0.430(0.226–0.820)	0.010*
Age	2.122(1.171–3.844)	0.013		
Ann Arbor stage	2.342(1.306–4.201)	< 0.001		
B symptoms	1.347(0.768–2.362)	0.298		
ECOG score	1.757(1.042–3.015)	0.041		
Cell origin	0.557(0.301–1.033)	0.063		
Bone marrow invasion	1.291(0.579–2.882)	0.533		
Extranodal invasion	0.230(0.358–1.259)	0.214		
LDH	2.538(1.442–4.466)	0.001		
PNI	0.270(0.135–0.540)	< 0.001	0.303(0.137–0.669)	0.003*
IPI	2.908(1.657–5.104)	< 0.001	2.159(1.206–3.863)	0.010*

*Representative *P* < 0.05.

Through univariate analysis of progression-free survival explored by COX regression, it was found that gender, age, Ann Arbor stage, ECOG, LDH level, PNI, and IPI were factors influencing the progression-free survival of DLBCL patients. Multivariate analysis showed that gender, age, and PNI (all *P* < 0.05) were all independent risk factors for disease progression, as shown in Table 7.

**TABLE 7:** Univariate and multivariate analysis (PFS) of prognostic factors in 171 patients with DLBCL.

Variables	Univariate(PFS)		Multivariate(PFS)	
	HR(95%CI)	*P*	HR(95%CI)	*P*
Gender	0.514(0.227–0.953)	0.035	0.485(0.258–0.912)	0.025*
Age	2.243(1.253–4.015)	0.007	1.920(1.065–3.462)	0.047*
Ann Arbor stage	2.133(1.195–3.084)	0.010		
B symptoms	1.347(0.768–2.362)	0.298		
ECOG score	1.757(1.042–3.015)	0.041		
Cell origin	0.544(0.677–2.094)	0.545		
Bone marrow invasion	0.943(0.424–2.096)	0.943		
Extranodal invasion	0.557(0.295–1.051)	0.071		
LDH	2.219(1.262–3.902)	0.006		
PNI	0.315(0.158–0.630)	0.001	0.364(0.160–0.829)	0.016*
IPI	2.405(1.377–4.201)	0.002		

*Representative *P* < 0.05.

From the series of analyses performed, it was found that IL-2R > 1,202 U/mL and PNI ≤ 44.65 are independent prognostic factors for adverse OS and PFS in newly diagnosed DLBCL, offering complementary prognostic information to traditional clinical factors.

## 4 Discussion

### 4.1 IL-2R is related to the immune function and nutritional status of DLBCL patients

Research suggests that IL-2 is a multifunctional factor involved in T cell growth and proliferation. Specifically, Miyazaki et al. identified three distinct IL-2 signaling pathways mediated by bcl-2, c-myc, and lck that cooperate to promote hematopoietic cell proliferation (16), laying a foundation for understanding the functional links between IL-2/IL-2R and immune cell regulation. Beyond this, IL-2/IL-2R has also been reported to participate in the regulation of cell apoptosis and death, as well as the differentiation of Th cells (17, 18), which further links IL-2/IL-2R to immune function. In this study, there was a strong correlation between IL-2R and age. In the entire cohort, as the age increased, the proportion of patients with high IL-2R expression increased. At the same time, Spearman correlation analysis demonstrates a significant inverse correlation between peripheral blood lymphocyte count and serum IL-2R levels in newly diagnosed DLBCL patients (Figure 2D), with a distinct linear trend: as lymphocyte counts increase, IL-2R levels exhibit a gradual and consistent downward tendency. Lymphocytes serve as core components of the bodyd consistent downward tendency. Lympalyscal roles in recognizing malignant cells, mediating antitumor immune responses, and maintaining immune surveillance. The relatively lower lymphocyte counts in patients with high IL-2R levels indicate a relative reduction in immune functional capacity. This observation is biologically plausible: elevated IL-2R may reflect excessive activation of immune cells, leading to a decrease in the number of functional lymphocytes available for antitumor defense, thereby weakening the body functionalto combat DLBCL progression. CRP is a classic inflammatory marker. Faramand classified DLBCL patients into low-risk, medium-risk, and high-risk groups according to the levels of CRP and ferritin, and observed that an increase in CRP often indicates a poor prognosis for DLBCL patients after receiving chimeric antigen receptor T-cell immunotherapy (CAR-T) treatment (19). A meta-analysis also strongly demonstrated that CRP can predict the survival prognosis of DLBCL patients, and an elevated CRP indicates a poor prognosis (20). In this study, the baseline level of IL-2R was correlated with CRP. Patients with a high level of IL-2R often had a high level of CRP. The nutritional status was reflected by BMI, albumin level, and total cholesterol level. There was no correlation between the IL-2R level and the patient’s BMI, but there was a correlation with the albumin and total cholesterol levels. This suggests that patients with a better immune status have a lower level of IL-2R in their bodies. Considering the relationships between immune function, nutritional status, and the IL-2R level, it can be preliminarily concluded that patients with a high IL-2R level often have immunodeficiency and poor nutritional status.

### 4.2 PNI is associated with serum inflammatory markers in DLBCL patients

PNI is a comprehensive indicator considering both nutritional and immune aspects. Correlation analysis showed that PNI decreases with increasing age, while BMI is weakly positively correlated with PNI (Figure 3B). This weak correlation is biologically plausible: BMI primarily reflects body mass composition, whereas PNI integrates serum albumin (a direct nutritional marker) and lymphocyte count, resulting in an indirect association between BMI and PNI. In the field of hematology, serum inflammatory markers such as CRP, LDH, and ferritin are closely associated with disease prognosis (19, 21). Statistically, this study found significant correlations between PNI and CRP, LDH, and ferritin (all *P* < 0.001); linear regression analysis revealed that PNI tends to decrease as CRP, LDH, and ferritin levels increase. Clinically, therefore, DLBCL patients with low PNI are highly likely to have concomitant high inflammatory responses. Thus, PNI may serve as an auxiliary reference for assessing DLBCL patients’ nutritional-immune status and stratifying prognostic risk, while its role in guiding individualized treatment awaits further prospective clinical validation.

### 4.3 Association between IL-2R and prognosis of DLBCL patients

In the survival analysis, it is clear that both the OS and PFS of the low-IL-2R group are superior to those of the high-IL-2R group. Due to the high heterogeneity of DLBCL, we divided DLBCL patients into more subgroups to compare the impact of IL-2R on the overall survival rate of these subgroups. With the cut - off value remaining at 1202 U/mL, we obtained the result that patients with a high IL-2R level had a worse survival outcome in subgroups including those with age > 60 years old, those with gastrointestinal location, non-GCB subtype, and Ann Arbor stage III–IV. Multivariate analysis indicated that IL-2R > 1202 U/mL is an independent influencing factor for the OS and PFS of DLBCL patients. We believe that IL-2R is related to the survival prognosis of DLBCL. The higher the level of IL-2R, the more harmful it is to the body, and it has a wide range of practical applications and can be used in multiple subgroups. Regarding the underlying immunomodulatory mechanisms linking high IL-2R to poor prognosis, we propose two plausible explanations supported by existing evidence. First, T cell exhaustion (TEX) may contribute to impaired anti-tumor immunity in patients with high IL-2R. Chronic antigen stimulation by tumors or persistent inflammation can induce TEX, characterized by reduced effector function and increased expression of inhibitory receptors, which weakens the body’s ability to eliminate malignant cells (22, 23). Notably, elevated IL-2R has been associated with enhanced TEX in hematological malignancies, as sustained IL-2/IL-2R signaling can drive excessive T cell activation and subsequent exhaustion (23). This aligns with our finding that high IL-2R correlates with lower lymphocyte counts—an indirect reflection of compromised immune effector cell function. Second, enhanced regulatory T cell (Treg) differentiation mediated by IL-2/IL-2R signaling may be another critical mechanism. Tregs play a key role in suppressing anti-tumor immune responses, and IL-2 is a central cytokine promoting Treg proliferation and functional stability (24, 25). Elevated IL-2R levels may amplify IL-2 signaling, leading to increased Treg accumulation in the tumor microenvironment and subsequent inhibition of effector T cell activity (26). This mechanism is particularly relevant in DLBCL, as increased Treg infiltration has been consistently linked to poor prognosis (27). While both mechanisms are supported by preclinical and clinical evidence, the exact contribution of TEX versus Treg differentiation to the adverse outcomes of high IL-2R DLBCL patients remains to be clarified. Future studies focusing on immune cell subsets and their functional status in relation to IL-2R levels will help validate these hypotheses and provide deeper insights into the immunopathological basis of DLBCL

### 4.4 Association between PNI and prognosis of DLBCL patients

According to the results of our study, patients with a PNI > 44.5 have a longer OS and PFS. This result can be observed in subgroups including those with age > 60 years old, non-GCB subtype, and Ann Arbor stage III–IV. In the gastrointestinal group, no significant survival difference was observed between the two groups. We believe that this is due to the small number of cases, making it difficult to obtain effective results. Through both univariate and multivariate analyses, it can be concluded that PNI ≤ 44.65 is an independent influencing factor for the poor prognosis of newly diagnosed DLBCL patients. PNI was originally a prognostic nutritional index used by Buzby et al. to evaluate gastrointestinal surgery (28). Today, PNI is no longer limited to postoperative evaluation. Subsequently, researchers found that PNI is applicable in multiple disease areas. Among 337 esophageal cancer patients, compared with those with a high PNI, patients with a low PNI had a significantly poorer overall survival. Meanwhile, it was also significantly associated with the lymphocyte infiltration status and the CD8-positive cell count (29). A study on migraine in the neurology department suggests that a low PNI is associated with severe headache or migraine. People who are prone to headaches tend to consume relatively less protein, lipids, folic acid, and various vitamins (30). The extensive application of PNI in multiple disciplines also validates the importance of immune function and nutritional status. This indicates that in the clinical field, a comprehensive consideration is needed when evaluating diseases. Nutritional status plays a crucial role in patients’ fight against diseases. Conducting an all-round assessment of DLBCL patients at an early stage and actively formulating individualized treatment plans will help provide patients with more professional and comprehensive treatment options.

### 4.5 Limitations of the research

This study also has some limitations. In terms of samples, only 171 patients were included, with a small sample size and possible homogeneity issues, which are prone to sampling errors and affect the universality and accuracy of the results. At the data level, the retrospective data bias may interfere with the analysis results. The research design lacks prospective verification, and the single treatment regimen makes it difficult to accurately capture the dynamic changes of the disease and evaluate the differences in prognostic factors under different treatments. In the future, multi-center studies can be carried out in collaboration with multiple medical centers, with prospective data collection covering populations from different regions and socioeconomic backgrounds, so as to reduce sampling errors, enhance the universality of results, and provide more solid evidence for the precise prognostic assessment of DLBCL.

## 5 Conclusion

IL-2R and PNI have good predictive value in the prognostic assessment of newly diagnosed DLBCL patients. IL-2R > 1, 202 U/mL and PNI ≤ 44.65 are independent risk factors for poor prognosis in patients, and are applicable to various subtypes of DLBCL.

## Data Availability

The raw data supporting the conclusions of this article will be made available by the authors, without undue reservation.
